# Genome-wide gene expression analysis of anguillid herpesvirus 1

**DOI:** 10.1186/1471-2164-14-83

**Published:** 2013-02-06

**Authors:** Steven J van Beurden, Ben PH Peeters, Peter JM Rottier, Andrew J Davison, Marc Y Engelsma

**Affiliations:** 1 Central Veterinary Institute, part of Wageningen UR, P.O. Box 65, Lelystad, AB, 8200, The Netherlands; 2 Department of Infectious Diseases and Immunology, Faculty of Veterinary Medicine, Utrecht University, P.O. Box 80.165, Utrecht, TD, 3508, The Netherlands; 3 MRC–University of Glasgow Centre for Virus Research, Glasgow, G11 5JR, UK; 4 Current address: Immunology-Vaccinology (B43b), Department of Infectious and Parasitic Diseases, Faculty of Veterinary Medicine, University of Liège, Liège, 4000, Belgium

**Keywords:** Anguillid herpesvirus 1, Temporal gene expression, Kinetic class, RT-qPCR

## Abstract

**Background:**

Whereas temporal gene expression in mammalian herpesviruses has been studied extensively, little is known about gene expression in fish herpesviruses. Here we report a genome-wide transcription analysis of a fish herpesvirus, anguillid herpesvirus 1, in cell culture, studied during the first 6 hours of infection using reverse transcription quantitative PCR.

**Results:**

Four immediate-early genes – open reading frames 1, 6A, 127 and 131 – were identified on the basis of expression in the presence of a protein synthesis inhibitor and unique expression profiles during infection in the absence of inhibitor. All of these genes are located within or near the terminal direct repeats. The remaining 122 open reading frames were clustered into groups on the basis of transcription profiles during infection. Expression of these genes was also studied in the presence of a viral DNA polymerase inhibitor, enabling classification into early, early-late and late genes. In general, clustering by expression profile and classification by inhibitor studies corresponded well. Most early genes encode enzymes and proteins involved in DNA replication, most late genes encode structural proteins, and early-late genes encode non-structural as well as structural proteins.

**Conclusions:**

Overall, anguillid herpesvirus 1 gene expression was shown to be regulated in a temporal fashion, comparable to that of mammalian herpesviruses.

## Background

Herpesvirus gene expression occurs in a temporally regulated fashion involving three major gene sets [1,2]. The first set consists of the immediate-early genes, which are defined as those that are transcribed in the absence of *de novo* protein synthesis. These genes regulate the subsequent expression of other genes. The next set, the early genes, encodes the enzymes involved in nucleotide metabolism and replication of the viral genome, and several envelope glycoproteins. The final set, the late genes, requires viral DNA synthesis for expression and encodes viral structural proteins. There is usually no clear boundary between early and late expression, and an intermediate leaky-late or early-late group has been proposed. Although this widely-used classification of herpesvirus genes may not fully reflect the subtle regulation of viral gene expression in infected cells, it helps understanding of the viral replication cycle, gene functions, virus-host interactions and possibilities for control of disease.

Herpesvirus genes have traditionally been classified kinetically on the basis of individual expression studies in cell culture [3]. Application of specific chemicals to inhibit selectively the expression of early and/or late genes has contributed to the classification and functional characterization of genes. More recently, genome-wide microarray and reverse transcription quantitative (RT-q)PCR expression studies have been performed for several mammalian herpesviruses in the family *Herpesviridae*[4-11]. However, little is known about expression in members of the family *Alloherpesviridae*, which infect amphibians and fish.

For channel catfish virus (ictalurid herpesvirus 1, IcHV1) the expression kinetics of a limited number of open reading frames (ORFs), namely ORF3, ORF5, ORF5/6, ORF6, ORF8A/9, ORF9, ORF12/13, ORF39 and ORF46 [12-14], has been studied in cell culture by northern blot analyses. Transcriptional regulation of all 14 ORFs in the terminal direct repeat of the genome has been analysed by northern blot analysis in cell culture [15] and by RT-PCR and Southern blot analysis *in vivo*[16]. For this small number of IcHV1 ORFs, temporal expression patterns similar to that of mammalian herpesviruses have been demonstrated. In addition, transcription in cell culture of 20 ORFs in koi herpesvirus (cyprinid herpesvirus 3, CyHV3) has been demonstrated by RT-PCR [17].

Anguillid herpesvirus 1 (AngHV1) causes a haemorrhagic disease and is associated with increased mortality rates in the Japanese eel (*Anguilla japonica*) and European eel (*Anguilla anguilla*) [18,19]. In this first report on genome-wide transcription of an alloherpesvirus, we characterized expression in cell culture of AngHV1 ORFs that are predicted to encode functional proteins by using RT-qPCR, which permits sensitive quantitation of specific RNAs [20,21]. Expression of early and late ORFs was selectively blocked by a protein synthesis inhibitor, and expression of late ORFs was inhibited by a viral DNA polymerase inhibitor. The genes were classified on the basis of their expression patterns in the presence of these inhibitors. We identified four putative immediate-early ORFs, and – on the basis of their transcription profiles – clustered all but three of the remaining 125 ORFs into early, early-late and late gene categories. The analysis relied on an AngHV1 map [GenBank:FJ940765.3] that is a substantial improvement on the original map derived from bioinformatic analysis of the complete genome sequence [22]. The updated map was derived from a deep-sequencing transcriptome study [23], and included redefined ORFs and experimentally supported polyadenylation (polyA) sites. The deep sequencing study also showed that, overall, 98.5% of late transcription of AngHV1 ORFs is directed by the sense DNA strand.

## Results and discussion

One-day old eel kidney 1 (EK-1) cell monolayers [24] in 6-well plates were pre-incubated with either virus growth medium (VGM), VGM containing protein synthesis inhibitor cycloheximide (CHX), or VGM containing viral DNA polymerase inhibitor phosphonoacetic acid (PAA). After the pre-incubation, the cells were infected with the Dutch AngHV-1 isolate CVI500138 [25] at a multiplicity of infection (MOI) of 10 50% tissue culture infective dose (TCID_50_)/cell in the appropriate VGM. RNA was extracted at t = 0, 1, 2, 4, and 6 h post infection (hpi). Potential contaminating DNA was removed, polyA RNA was extracted and reverse transcribed into cDNA. SYBR Green quantitative (q)PCR reactions were carried out for all AngHV1 ORFs, taking a reaction for the reference β-actin gene of European eel [26] into account in every qPCR run for each time point. Based on the efficiency of amplification (E) for each primerset, relative expression ratio (R) [7,27], change in relative gene expression (R_Δ_), inhibitory effect of CHX at t = 4 and t = 6 hpi (R_i-CHX_), and inhibitory effect of PAA at t = 6 hpi (R_i-PAA_) were calculated.

### Reproducibility

Experiments in which cells were untreated were carried out three times, and experiments in which cells were treated with PAA or CHX were carried out twice. The β-actin reference gene Ct-values were highly reproducible; the mean value ± standard deviation of all measurements (n = 656) of all experiments was 16.73 ± 2.14 (coefficient of variation = 12.8%).

Relative expression ratios were calculated independently for each experiment, and the mean and standard deviation values were subsequently calculated for all ORFs and time points. The average coefficient of variation for R_i-CHX_ for the non-inhibited ORFs at t = 4 and t = 6 hpi was 21.8% and 22.7%, respectively. The average coefficient of variation for R_i-PAA_ for all ORFs at t = 6 hpi was 16.8%.

### Families of 3’-coterminal transcripts

Although herpesvirus genes generally have individual promotors, it is not uncommon for a single polyA site to be shared by two or more genes arranged as a 3’-coterminal family [28]. In the deep-sequencing transcriptome study of AngHV1 [23], 32 such families were identified. With regard to the current study, the RT-qPCR signals detected for the 58 ORFs that are not in 3’-coterminal families and the 32 ORFs that are located at the 5’-ends of 3’-coterminal families can be considered accurate. However, signals detected for the remaining 36 ORFs might be mixed with those from upstream ORFs, and a more tentative interpretation is implicit in the results discussed below.

### AngHV1 gene expression

Gene expression was detected at some time point for all predicted functional protein-coding ORFs in the AngHV1 genome. In general, levels were absent or very low at t = 1 and t = 2 hpi and increased exponentially during the monitored period. ORF1, ORF6A, ORF127 and ORF131 were the first genes becoming detectable, they already showed significant expression at t = 2 hpi. Maximum expression was measured at t = 6 hpi for all ORFs, except for ORF1, ORF86 and ORF130, which peaked at t = 4 hpi.

**Figure 1:**
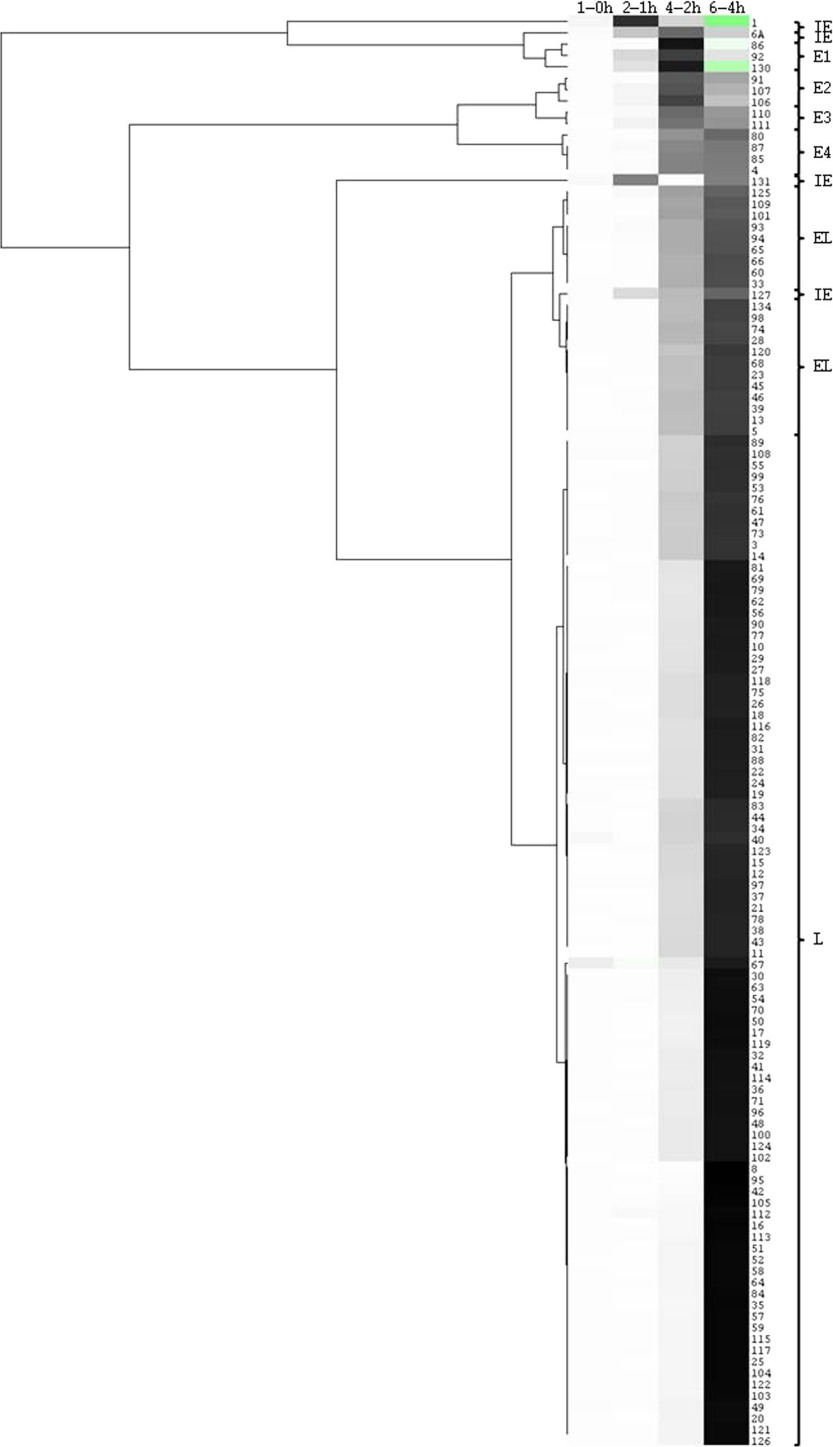
**Cluster analysis of AngHV1 ORFs on the basis of R**_**Δ**_**-values.** Complete linkage hierarchical clustering analysis of the R_Δ_-values of all AngHV1 ORFs for the time points R_1_-R_0_, R_2_-R_1_, R_4_-R_2_ and R_6_-R_4_. Java TreeView pixel settings were: contrast = 1.0, positive = black, zero = white, negative = green. Clades were flipped as to visualize genes in order of expression from top to bottom. Accolades indicate clusters of genes with comparable gene expression profiles, corresponding to the groups shown in Figure 2.

**Figure 2:**
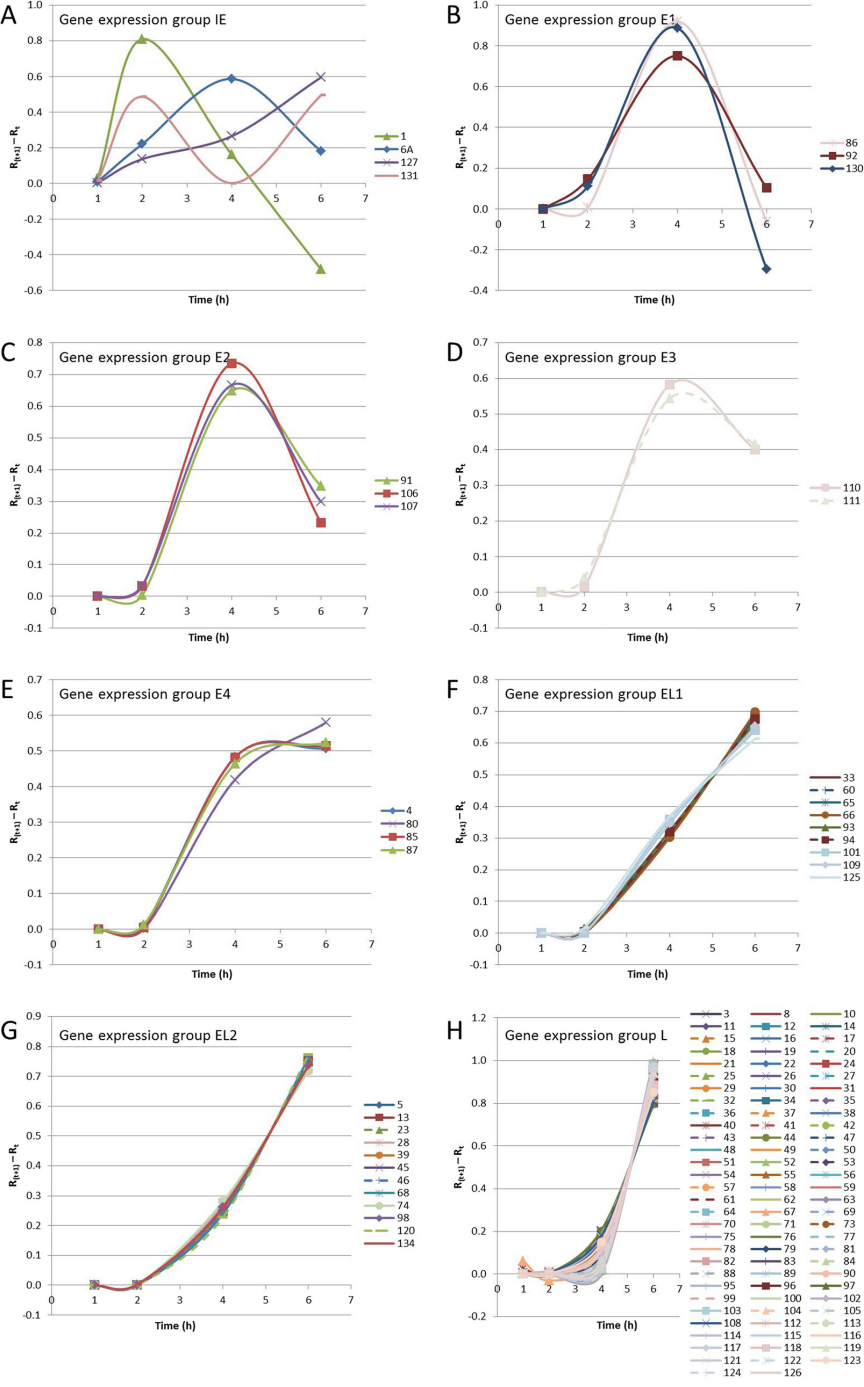
**Plots of groups of AngHV1 ORFs with comparable R**_**Δ**_**-value-based gene expression profiles.** All AngHV1 genes were grouped on the basis of cluster analysis of their gene expression profiles based on their R_Δ_-values over the four time intervals. Gene expression group names correspond to clustering in Figure 1. Expression profile graphs of ORFs potentially compromised by 3’-coterminality are dashed.

The net change in transcription at each time interval or R_Δ_ was calculated for each gene (Additional file 1: Table S1). The genes were clustered based on their R_Δ_-values, and ten groups were distinguished (Figure 1). R_Δ_-graphs are shown for all gene groups (Figure 2), with the unique expression kinetics of ORF1, ORF6A, ORF127 and ORF131 combined in one (Figure 2A, group IE). The R_Δ_-graphs of the upstream located 3’-coterminal ORFs are represented by dashed lines.

The R_Δ_ of the 12 members of groups E1-4 is characterized by a very low value at R_2_-R_1_, a steep increase between R_2_-R_1_ and R_4_-R_2_, and a decrease or stabilization at R_6_-R_4_ (Figure 2B-E). Differentiation between the E groups is based on the R_Δ_-value at R_6_-R_4_, which varies from practically zero (group E1) to more or less similar at R_4_-R_2_ and R_6_-R_4_ (group E4).

The R_Δ_ of members of groups EL1 and EL2 is characterized by a very low value at R_2_-R_1_, and a more or less linear increase between R_2_-R_1_ and R_6_-R_4_ (Figure 2F-G). The 9 members of group EL1 show a slightly earlier but less steep increase of R_Δ_ than the 12 members of group EL2. Both members of the ORF109 gene family (ORF109 and ORF125) belong to group EL1, with ORF125 being identified as a low-abundant envelope protein [29].

Most of the genes fall into group L (n = 89). The R_Δ_-value is zero or very low at R_2_-R_1_, very low or low at R_4_-R_2_, and almost maximal at R_6_-R_4_ (Figure 2H). Group L comprises both members of the ORF3 gene family (ORF3 and ORF14), both members of the ORF11 gene family (ORF11 and ORF12), three members of the ORF13 gene family (ORF16, ORF17 and ORF24), three members of the ORF68 gene family (ORF69, ORF70 and ORF73), both members of the deoxyguanosine kinase (dGK) gene family (ORF79 and ORF123), and three members of the tumour necrosis factor receptor (TNFR) gene family (ORF108, ORF121 and ORF124).

In the analysis described above, we followed the RT-qPCR-based approach used to study gene expression by Tombácz *et al.*[7] in pseudorabies virus (PRV; a mammalian herpesvirus in subfamily *Alphaherpesvirinae*, family *Herpesviridae*). In general, gene expression profiles are highly comparable for AngHV1 and PRV. The AngHV1 and PRV immediate-early genes and the PRV large latency transcript demonstrate unique expression profiles, genes in group E show maximum increase of gene expression between t = 2 and t = 4 hpi, genes in group L show maximum increase of gene expression between t = 4 and t = 6 hpi, and genes in group EL demonstrate an intermediate expression profile. AngHV1 comprises about twice as much ORFs as PRV, which mainly add up to the EL and L groups.

### Immediate-early transcripts of AngHV1

**Figure 3:**
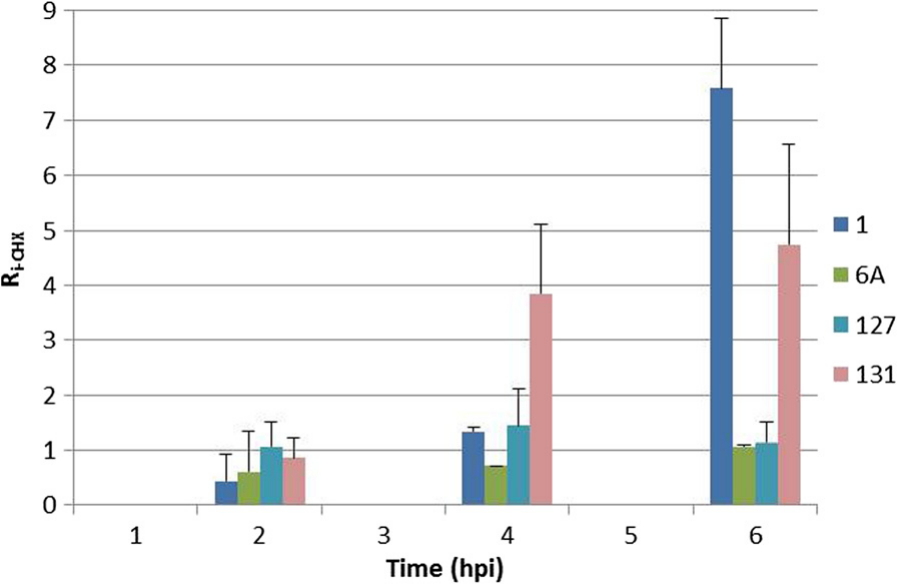
**Relative inhibition of immediate-early gene expression in the presence of CHX (R**_**i-CHX**_**).** Inhibitory effect of CHX on gene expression (R_i-CHX_) at t = 2, 4 and 6 hpi. Only the four ORFs that exhibited no significant inhibition of gene expression are shown (ORF1, ORF6A, ORF127 and ORF 131). Standard deviations of R_i-CHX_-values were calculated from two independent experiments and are indicated by error bars.

Inhibition of *de novo* protein synthesis by CHX resulted in an inhibition of relative gene expression (R_i-CHX_) of >66% at t = 4 and of >91% at t = 6 hpi for all but 4 ORFs. ORF6A and ORF127 showed almost no inhibition at t = 4 and t = 6 hpi (Figure 3). ORF1 demonstrated an increase in relative expression of 1.3 at t = 4 and of 7.6 at t = 6 hpi. ORF131 exhibited an increase in relative expression of 3.8 at t = 4 and of 4.7 at t = 6 hpi.

In cells not treated with inhibitors, these 4 immediate-early ORFs showed unique expression profiles that were clearly distinct from those of other ORFs (group IE). No imputed amino acid sequence similarities with ORFs in other alloherpesviruses were detected [22]. ORF127 has been shown to encode a low-abundant structural tegument protein [29].

### Early and early-late transcripts of AngHV1

Inhibition of viral DNA polymerase by PAA resulted in an inhibition of gene expression (R_i-PAA_) at t = 6 hpi for all but two ORFs (Additional file 2: Table S2). In the presence of PAA, the relative expression of ORF1 was doubled, and that of ORF6A was not affected. Table 1 shows the mean and standard deviations of R_i-PAA_-values in the presence of PAA at t = 6 hpi for the 61 ORFs of AngHV1 for which a putative function has been described (listed on the basis of their R_i-PAA_-values), with the kinetic classes of ORFs potentially compromised by 3’-coterminality marked with asterisks.

**Table 1:** **Expression of selected AngHV1 genes, sorted on the basis of their R**_**i-PAA**_**-values at t = 6 hpi**

**ORF**^**a**^	**Mean**^**b**^	**STDEV**^**c**^	**Kinetic class**^**d**^	**Function**^**e**^
1	1.800	0.475	Immediate early	
6A	1.059	0.115	Immediate early	
127	0.690	0.120	Immediate early	
87	0.609	0.019	Early	Serine-threonine protein kinase
39	0.529	0.057	Early	Tegument protein
14*	0.522	0.022	Early	Tegument protein
101	0.519	0.089	Early	Tumour necrosis factor receptor domain
131	0.493	0.016	Immediate early	
15*	0.462	0.074	Early	Guanosine triphosphatase
55	0.457	0.004	Early	DNA polymerase
10	0.442	0.156	Early	ATPase subunit of terminase
18*	0.429	0.038	Early/Late	Tegument protein
67	0.427	0.078	Early/Late	Major glycoprotein
66	0.417	0.036	Early/Late	Envelope protein
116	0.359	0.002	Early/Late	Ribonucleotide reductase (large subunit)
123	0.340	0.039	Early/Late	Deoxyguanosine kinase
78	0.329	0.032	Early/Late	Envelope protein
37*	0.316	0.041	Early/Late	DNA helicase
5	0.313	0.064	Early/Late	Deoxyuridine thriphosphatase
108	0.308	0.011	Early/Late	Envelope protein
77*	0.287	0.004	Early/Late	Thymidylate kinase
38	0.271	0.023	Early/Late	Tegument protein
90	0.264	0.007	Early/Late	Nucleoside diphosphate kinase
83	0.263	0.073	Early/Late	Large tegument protein
21	0.262	0.058	Early/Late	Primase
29	0.259	0.025	Early/Late	Uracil-DNA glycosylase
19*	0.253	0.005	Early/Late	Tegument protein
75	0.246	0.034	Early/Late	Thymidylate synthetase
34	0.242	0.028	Late	Tegument protein
125	0.222	0.024	Late	Envelope protein
43*	0.206	0.072	Late	Tegument protein
32*	0.201	0.051	Late	Tegument protein
71	0.199	0.023	Late	Envelope protein
81*	0.196	0.013	Late	Tegument protein
79	0.188	0.027	Late	Deoxyguanosine kinase
40	0.185	0.038	Late	Tegument protein
20*	0.179	0.002	Late	Tegument protein
124*	0.174	0.012	Late	Tumour necrosis factor receptor domain
119	0.170	0.028	Late	Dihydrofolate reductase
17*	0.162	0.020	Late	Tegument protein
100	0.160	0.034	Late	Capsid protein
36*	0.154	0.071	Late	Capsid triplex protein 2
114	0.153	0.025	Late	Tegument protein
26	0.147	0.062	Late	Tegument protein
24	0.145	0.076	Late	Tegument protein
96	0.124	0.029	Late	Ribonucleotide reductase (small subunit)
30	0.110	0.057	Late	Tegument protein
49	0.090	0.013	Late	Envelope protein
16	0.079	0.023	Late	Tegument protein
115	0.070	0.013	Late	Envelope protein
35*	0.068	0.011	Late	Tegument protein
48	0.068	0.015	Late	Capsid protein
57*	0.066	0.022	Late	Capsid protease-and-scaffolding protein
25*	0.063	0.016	Late	Interleukin 10 homolog
51	0.063	0.026	Late	Envelope protein
103	0.057	0.012	Late	Tegument protein
104*	0.050	0.007	Late	Major capsid protein
126	0.031	0.008	Late	Capsid protein
42*	0.024	0.001	Late	Capsid triplex protein 1
8	0.010	0.004	Late	Envelope protein
95	0.010	0.000	Late	Envelope infectious salmon anaemia virus haemagglutinin-esterase protein

^a^ ORF numbering corresponds to van Beurden *et al.*[22]; only ORFs for which a putative function has been predicted are shown; ORFs from which the data are potentially compromised by 3’-coterminality are marked with asterisks.

^b^ Mean R_i-PAA_-values were calculated from two independent experiments.

^c^ Standard deviations of R_i-PAA_-values were calculated from two independent experiments.

^d^ Immediate-early genes were classified on the basis of CHX inhibition experiments; boundaries between early, early-late and late genes are explained in the text.

^e^ Gene functions were predicted based on similarity with known functional protein sequences [22], and on AngHV1 structural protein analyses by mass spectrometry [29].

Inhibition of gene expression by PAA ranges from about 30% for ORF87 to 99% for ORF95. A comparable range was described previously for PRV [7]. The ORFs are classified into kinetic classes on the basis of their R_i-PAA_-values, with arbitrarily set threshold values. The typical early gene ORF10, encoding the ATPase subunit of terminase, was chosen as the last early gene, and ORF18, encoding a structural tegument protein, as the first early-late gene. ORF75, encoding thymidylate synthetase, was denoted as the last of the early-late genes, and ORF34, encoding a structural tegument protein, as the first of the late genes. There was no clear boundary between early, early-late, and late genes based on their R_i-PAA_-values.

A total of 17 early genes were identified (Additional file 2: Table S2). Almost half of these showed a distinct expression profile in the untreated samples (members of groups E1-4), characterized by the largest increase of R_Δ_ between R_2_-R_1_ and R_4_-R_2_. Among the early genes are those involved in DNA replication (ORF55) and DNA packaging (ORF10), genes encoding a guanosine triphosphatase (GTPase) (ORF15), a serine-threonine protein kinase (ORF87), a protein encoding a TNFR domain (ORF101), and two low-abundant tegument proteins (ORF14 and ORF39). Early genes identified for IcHV1 included the thymidine kinase gene (IcHV1 ORF5), a protein kinase (IcHV1 ORF14), and two putative membrane proteins (IcHV1 ORF6 and IcHV1 ORF8) [14,15].

A total of 37 early-late genes were identified, with inhibition of expression values intermediate between those of early and late genes (Additional file 2: Table S2). In samples not treated with inhibitors, 4 of the early-late genes were grouped into one of the E groups, and 10 genes were classified in one of the EL groups. Functions have been predicted for 17 of the early-late genes (Table 1). These consist of 9 genes encoding non-structural proteins and 8 encoding structural proteins. Among the non-structural group are proteins involved in DNA replication (ORF21 and ORF37) and enzymes involved in nucleic acid metabolism. Among the structural early-late gene proteins are 4 tegument and 4 envelope proteins, but no capsid proteins. For IcHV1, a membrane protein (IcHV1 ORF7), a tegument protein (IcHV1 ORF11) and a zinc-binding protein (IcHV1 ORF12) were shown to specify both early and late transcripts [15]. Our findings are in general accordance with the properties of proteins encoded by early and early-late genes of mammalian herpesviruses.

### Late transcripts of AngHV1

A total of 68 late genes were identified (Additional file 2: Table S2), 6 of which were clustered into one or other of the EL groups, and 62 of which were grouped in the L group. Functions have been assigned to 33 of the late gene proteins (Table 1), 28 of which involve roles as structural proteins. All 7 capsid proteins, as well as 14 tegument proteins and 7 envelope proteins, were identified as being encoded by late genes. Similarly, the three late genes identified for IcHV1 encode the major capsid protein (IcHV1 ORF39) and two membrane proteins (ORF10 and ORF46) [14,15]. Two of the putative host-immunomodulatory proteins of AngHV1, namely an interleukin 10 homolog encoded by ORF25 [30] and a protein containing a TNFR domain encoded by ORF124 [22], were also identified as being encoded by late genes.

### Location of expression in the AngHV1 genome

**Figure 4:**
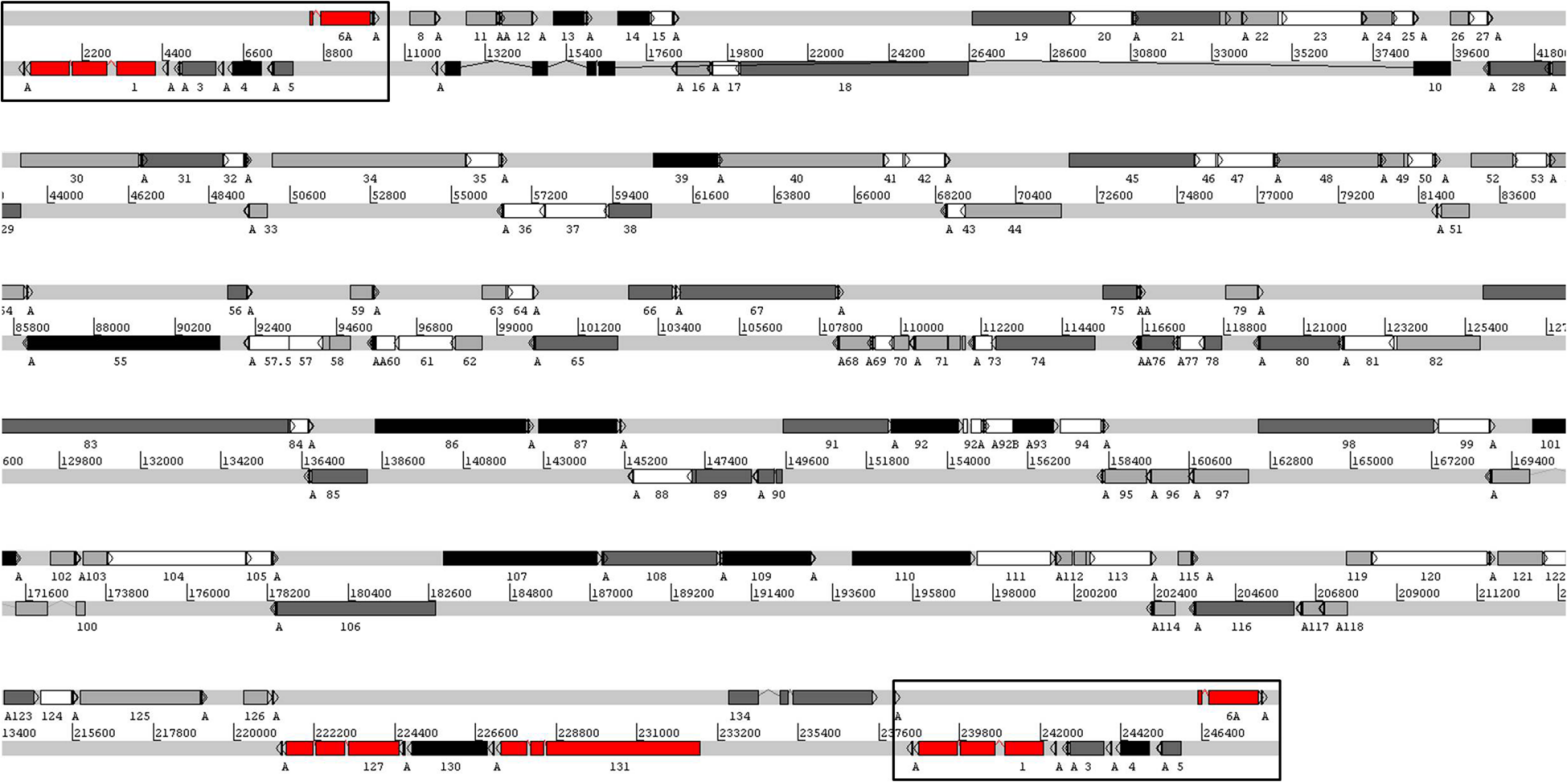
**Lay-out of AngHV1 ORFs showing kinetic class on basis of the R**_**i-PAA**_**-analysis at t = 6 hpi.** The gene layout of AngHV1 is based on [22] updated by van Beurden *et al.*[23]. Both strands are shown with the terminal repeats boxed. The locations of forward- and reverse-orientated ORFs are shown on the respective strands, labelled with the corresponding ORF number, and introns are depicted as thin lines connecting the exons. Identified polyA signals are depicted as small arrows on the respective strands, marked “A”. Kinetic class is indicated by colour, based on R_i-PAA_-values at t = 6 hpi, corresponding to Additional file 2: Table S2. Red = immediate early gene, black = early gene, dark grey = early/late gene, light grey = late gene, white = ORFs potentially compromised by 3’-coterminality. Analysis was not performed for ORF57.5, ORF92A and ORF92B, and hence kinetic class could not be determined.

A stringent summary of the kinetic classes of AngHV1 ORFs deduced from the R_i-PAA_-analysis at t = 6 hpi is shown in Figure 4, with the 36 ORFs potentially compromised by 3’-coterminality and the three ORFs for which no data were obtained left blank. The immediate-early ORFs are located within the terminal direct repeats (ORF1 and ORF6A) and near the right end of the unique region (ORF127 and ORF131). The two or three immediate-early genes of IcHV1 identified in a comparable way are also located in the terminal direct repeats [13-15]. The early, early-late and late genes are seemingly distributed randomly throughout the genome.

### Differences in expression kinetics of AngHV1 genes with and without inhibitors

In the present study, we classified the AngHV1 genes on the basis of their expression patterns in the presence of specific inhibitors selectively blocking the expression of early and/or late genes. This classification is not fully consistent with the clustering of genes on the basis of their expression profiles in the absence of an inhibitor. Whereas all immediate-early genes also showed unique expression profiles in cells not treated with inhibitors (IE group), and most late genes were expressed later than the other genes (L group), only half of the early and one-third of the early-late genes clustered in the E and EL groups, respectively. The latter finding may partly be explained by the incomplete blockage of the expression of late genes by the viral DNA polymerase inhibitor, but may also reflect the more complex regulation of viral gene expression during normal infection. The genes with marked differences in expression kinetics between both approaches are of interest for further functional characterization.

## Conclusions

We have studied the kinetics of gene expression in AngHV1 by genome-wide transcription analyses using RT-qPCR. Four immediate-early genes were identified on the basis of their unique gene expression profiles in cells not treated with inhibitors, and on the basis of their unchanged or increased gene expression in the presence of a protein synthesis inhibitor. Additional characterization of these genes is required to determine their potential roles in the regulation of gene expression. On the basis of inhibition of gene expression in the presence of a viral DNA polymerase inhibitor, 17 early genes, 37 early-late genes and 68 late genes were identified. This classification broadly complied with cluster analysis of the genes based on their changes in relative gene expression in untreated samples. Our findings on the broad functions of genes assigned to different kinetic classes are in accordance with those from a more limited exercise with IcHV1, and may be extrapolated to related alloherpesviruses. Comparable to mammalian herpesviruses, most of the early and early-late genes encode proteins involved in viral DNA replication, enzymes involved in nucleic acid metabolism, and several envelope glycoproteins. Most of the late genes encode structural proteins. Hence, we suggest that, despite the virtual absence of detectable genetic similarities between the herpesvirus families, fish herpesviruses of the family *Alloherpesviridae* exhibit patterns of temporally regulated gene expression that are similar to those of mammalian herpesviruses in the family *Herpesviridae*.

## Methods

### Cell culture

The Dutch AngHV1 isolate CVI500138 [25] was propagated in one-day old ~80% confluent eel kidney 1 (EK-1) cell monolayers [24]. The cells were cultured in monolayers in sterile plastic culture flasks (Falcon, BD Biosciences, Bedford, MA, USA) with virus growth medium (VGM), consisting of Leibovitz’s L-15 medium (Gibco, Invitrogen by Lifetechnologies, Carlsbad, CA, USA) with 2% (v/v) fetal bovine serum (FBS, Bodinco, Alkmaar, The Netherlands), 0.075% (w/v) NaHCO_3_, 2 mM L-glutamine (Gibco) and antibiotics (0.012% (w/v) kanamycin (Sigma-Aldrich, St. Louis, MO, USA) and 270 IE/ml penicillin G (Astellas Pharma, Tokyo, Japan)) at 26°C in a 5% CO_2_-incubator (Nuaire, Plymouth, MN, USA). For virus propagation on 6-well plates (Cellstar, Greiner bio-one, Frickenhausen, Germany), 0.26% instead of 0.075% (w/v) NaHCO_3_ was used.

### Virus infections

One-day old EK-1 monolayers in 6-well plates were washed once and pre-incubated for 1 h with either 5 ml of VGM, 5 ml of VGM containing 100 μg/ml protein synthesis inhibitor cycloheximide (CHX, Calbiochem, Merck, Darmstadt, Germany), or with 5 ml of VGM containing 400 μg/ml viral DNA polymerase inhibitor phosphonoacetic acid (PAA, Sigma-Aldrich). After the pre-incubation, the cells were infected with AngHV1 at a multiplicity of infection (MOI) of 10 50% tissue culture infective dose (TCID_50_)/cell in 2 ml of the appropriate VGM (t = 0). After a 30 min incubation period, cells were washed thrice with the appropriate VGM formulation and incubated for another 30 min, 1.5, 3.5 or 5.5 h. Infections were performed in triplicate with native VGM, and in duplicate for PAA- and CHX-containing VGM.

### mRNA extraction

RNA was extracted at t = 0 (non-infected control), 1, 2, 4, and 6 h post infection (hpi). Infected cells were washed once with the appropriate VGM formulation, and cells were lysed directly in the culture dish by adding 1 ml Trizol Reagent (Invitrogen). RNA was extracted according to manufacturer’s protocol, and dissolved in 200 μl RNase-free water (Sigma-Aldrich) with 2 μl RNaseOUT recombinant ribonuclease inhibitor (Invitrogen). RNA concentration was checked by spectrophotometry using a Nanodrop ND-1000 (Thermo Fisher Scientific, MA, USA).

Potential contaminating DNA was removed by treatment with DNase I (Roche, Basel, Switzerland). Twenty units of DNase I (2 μl) were added to a maximum of 50 μg total RNA (87.5 μl), 10 μl 10× Incubation buffer and 10 units RNaseOUT, and incubated for 20 min at 31°C at 550 rpm on a thermomixer (Eppendorf, Hamburg, Germany). DNase I was inactivated by adding 2 μl 0.2 M EDTA (pH 8.0) and heating for 10 min at 75°C at 550 rpm on a thermomixer. The concentration of DNase-treated RNA in the samples was checked by spectrophotometry.

PolyA RNA was extracted using an Oligotex mRNA kit (Qiagen, Hilden, Germany). In brief, 90 μl DNase-treated total RNA was used as starting material, following the manufacturer’s instructions for the spin-column protocol. PolyA RNA was finally dissolved in 2 × 75 μl of buffer OEB at 70°C. After cooling, 1.5 μl RNaseOUT was added and the sample was stored at −80°C.

### Reverse transcription

Reverse transcription (RT) was carried out using the TaqMan Reverse Transcriptase Reagent kit (Applied Biosystems by Lifetechnologies, Carlsbad, CA, USA). The total volume of RT mix prepared on ice was 100 μl per reaction, containing 10 μl RT buffer (10×), 22 μl MgCl_2_ (25 mM), 20 μl dNTP mixture (2.5 mM of each dNTP), 5 μl oligo dT (50 μM), 2 μl RNase Inhibitor (20 U/μl), 2.5 μl MultiScribe Reverse Transcriptase (50 U/μl), 23.5 μl DNase- and RNase-free water and 15 μl polyA RNA as template. The thermal profile of the RT program consisted of 10 min incubation at 25°C, 30 min RT at 48°C, 5 min RT inactivation at 95°C, and cooling to 4°C, and was performed in a 96-well GeneAmp PCR System 9700 (Applied Biosystems). For each polyA RNA sample, RT was carried out in duplicate, after which the cDNA was combined and diluted 1:5 in DNase- and RNase-free water and stored at −20°C.

### Quantitative PCR

The total volume of the SYBR Green quantitative (q)PCR mix was 20 μl per reaction, containing 10 μl SYBR Green PCR Mix (Applied Biosystems), 0.8 μl forward primer (10 μM), 0.8 μl reverse primer (10 μM), 0.25 μl Uracil-DNA Glycosylase (UDG, 5 U/μl, New England Biolabs, Ipswich, MA, USA), 3.15 μl super Q and 5 μl diluted cDNA template. The thermal profile of the SYBR Green qPCR program consisted of 10 min at 37°C, 10 min at 95°C, 40 cycles of 15 s at 95°C and 1 min at 60°C, followed by a dissociation stage from 60°C to 95°C at the end of the run, and was carried out in a 7500 Fast Real-Time PCR system (Applied Biosystems) under Standard 7500 conditions. In every qPCR run, for each time point reactions for the reference β-actin gene of European eel (*Anguilla anguilla*) were analysed, using the primer set described by Aroua *et al.*[26].

### Primers

Primers were designed for all AngHV1 ORFs, except for the newly predicted ORF57.5, ORF92A and ORF92B [23], using the Primer Express 3.0 program (Applied Biosystems) in the TaqMan and TaqMan MGB quantification mode, and evaluated using the Oligo Analyzer program v1.5 (Gene Link, Hawthorne, NY, USA). Primers are listed in Additional file 3: Table S3.

For all primer sets, specificity and efficiency were tested as described below. Viral DNA was extracted from purified AngHV1 virions [29] using the DNA blood mini kit (Qiagen) following the manufacturer’s protocol. Tenfold dilution series were prepared from AngHV1 DNA in buffer AE (Qiagen), and, for all primer sets, standard curves over four orders of magnitude were tested in triplicate. Primer sets were also checked for the formation of nonspecific reactions with host cell DNA. Primer sets were considered valid if primers did not form nonspecific products in non-template controls, and if standard curve slopes were at least −3.8. Average melting temperature (Tm-)values for each primer set were calculated from the efficiency tests. The efficiency of the β-actin reference gene primer set was determined to be 91.0% from tenfold dilution series over four orders of magnitude from cDNA from an infection trial with native VGM and VGM containing PAA or CHX at different time points post infection.

### Data analyses

Quantitative PCR data were analysed using the Sequence Detection Software version 1.4 program (Applied Biosystems) with the Auto baseline function and a threshold of 0.05. The means of the three Ct-values for each dilution point were calculated, and the efficiency of amplification (E) of each primer set was determined from the slope of the standard curves using the Microsoft Excel 2003 program (Microsoft, Redmond, WA, USA), using the formula:

$$E=10^{-1/\mathrm{slope}}$$

The relative expression ratio (R) was calculated for each sample using the formula modified from Pfaffl *et al.*[27] by Tombácz *et al.*[7]:

$$R=E_{\mathrm{sample}}{}^{\mathrm{Ct}\left[\mathrm{sample}\;\max\right]-\mathrm{Ct}\left[\mathrm{sample}\right]}/E_{\mathrm{reference}}{}^{\mathrm{Ct}\left[\mathrm{reference}\;\max\right]-\mathrm{Ct}\left[\mathrm{reference}\right]}$$

E_sample_ refers to the efficiency of amplification of a particular primer set, Ct[sample max] refers to the maximal Ct-value for that particular sample during the course of infection, Ct[sample] refers to any particular sample at a given time point, E_reference_ refers to the efficiency of amplification of the β-actin reference gene primer set, Ct[reference max] refers to the Ct-value for the β-actin reference gene corresponding to Ct[sample max], and Ct[reference] refers to the Ct-value for the β-actin reference gene corresponding to Ct[sample]. R-values, means and standard deviations were calculated for each sample using the Microsoft Excel 2003 program. The change in relative gene expression in untreated samples was calculated by subtracting the R-value of a given time point from the previous time point:

$$R_{\Delta }=R_{\left(t+1\right)}-R_{t}$$

Genes were clustered on the basis of their R_Δ_-values by using a complete linkage hierarchical clustering method with a centred correlation similarity metric, using the Cluster 3.0 computer program (M. Eisen, Stanford University, USA and M. de Hoon, University of Tokyo, Japan) and viewed with Alok Saldanha’s Java TreeView v1.1.6r2. R_Δ_-graphs of clustering genes were plotted using the Microsoft Excel program.

The inhibitory effect of CHX on gene expression (R_i-CHX_) at t = 4 and t = 6 hpi and the inhibitory effect of PAA on gene expression (R_i-PAA_) at t = 6 hpi were calculated using the formula of Pfaffl *et al.*[27].

## Competing interests

The authors declare that they have no competing interests.

## Authors’ contributions

SJvB designed the study, designed the primers, carried out the virus infections, mRNA extractions, RT, qPCR, data analyses and drafted the manuscript. BPHP and PJMR advised on the study design and manuscript. AJD provided the updated AngHV1 map (including 3’-ends), advised on results interpretation and manuscript. MYE coordinated the study, advised on the study design and manuscript. All authors read and approved the final manuscript.

## Supplementary Material

Additional file 1: Table S1 Net increase per time interval for each AngHV1 ORF (R_Δ_). Click here for file

Additional file 2: Table S2 Expression of AngHV1 genes, sorted on the basis of their R_i-PAA_-values at t = 6 hpi. Click here for file

Additional file 3: Table S3 Sequences and efficiency of qPCR primers targeting 126 AngHV1 ORFs. Click here for file
