# Supplementary material for: Genome-wide gene expression analysis of anguillid herpesvirus 1
Source: BMC Genomics. 2013 Feb 6;14:83. doi: 10.1186/1471-2164-14-83 (PMC3626852; doi:10.1186/1471-2164-14-83)
Supplement: Additional file 2: Table S2 — Expression of AngHV1 genes, sorted on the basis of their Ri-PAA-values at t = 6 hpi. [file 1471-2164-14-83-S2.docx]

**Table S2 – Expression of AngHV1 genes, sorted on the basis of their Ri-PAA-values at t = 6 hpi**

| ORF^a^ | Primerset^b^ | Mean^c^ | STDEV^d^ | Kinetic class^e^ | Function^f^ |
| --- | --- | --- | --- | --- | --- |
| 1 | AngHV-1.ORF1.45 | 1.800 | 0.475 | Immediate early |  |
| 6A | AngHV-1.ORF6.169 | 1.059 | 0.115 | Immediate early |  |
| 86 | AngHV-1.ORF86.115 | 0.923 | 0.037 | Early |  |
| 110 | AngHV-1.ORF110.140 | 0.731 | 0.098 | Early |  |
| 111* | AngHV-1.ORF111.141 | 0.722 | 0.062 | Early |  |
| 130 | AngHV-1.ORF130.160 | 0.695 | 0.028 | Early |  |
| 127 | AngHV-1.ORF127.157 | 0.690 | 0.120 | Immediate early |  |
| 93 | AngHV-1.ORF93.123 | 0.652 | 0.112 | Early |  |
| 13 | AngHV-1.ORF13.55 | 0.650 | 0.010 | Early |  |
| 92 | AngHV-1.ORF92.122 | 0.621 | 0.034 | Early |  |
| 87 | AngHV-1.ORF87.117 | 0.609 | 0.019 | Early | Serine-threonine protein kinase |
| 4 | AngHV-1.ORF4.48 | 0.579 | 0.112 | Early |  |
| 39 | AngHV-1.ORF39.76 | 0.529 | 0.057 | Early | Tegument protein |
| 14 | AngHV-1.ORF14.56 | 0.522 | 0.022 | Early | Tegument protein |
| 101 | AngHV-1.ORF101.131 | 0.519 | 0.089 | Early | Tumour necrosis factor receptor domain |
| 107 | AngHV-1.ORF107.137 | 0.508 | 0.047 | Early |  |
| 131 | AngHV-1.ORF131.176 | 0.493 | 0.016 | Immediate early |  |
| 15* | AngHV-1.ORF15.57 | 0.462 | 0.074 | Early | Guanosine triphosphatase |
| 55 | AngHV-1.ORF55.179 | 0.457 | 0.004 | Early | DNA polymerase |
| 109 | AngHV-1.ORF109.173 | 0.443 | 0.038 | Early |  |
| 10 | AngHV-1.ORF10.184 | 0.442 | 0.156 | Early | ATPase subunit of terminase |
| 3 | AngHV-1.ORF3.47 | 0.438 | 0.062 | Early/Late |  |
| 28 | AngHV-1.ORF28.68 | 0.437 | 0.108 | Early/Late |  |
| 56 | AngHV-1.ORF56.89 | 0.437 | 0.197 | Early/Late |  |
| 18 | AngHV-1.ORF18.60 | 0.429 | 0.038 | Early/Late | Tegument protein |
| 67 | AngHV-1.ORF67.99 | 0.427 | 0.078 | Early/Late | Major glycoprotein |
| 66 | AngHV-1.ORF66.98 | 0.417 | 0.036 | Early/Late | Envelope protein |
| 106 | AngHV-1.ORF106.136 | 0.412 | 0.214 | Early/Late |  |
| 45 | AngHV-1.ORF45.82 | 0.408 | 0.042 | Early/Late |  |
| 80 | AngHV-1.ORF80.109 | 0.399 | 0.078 | Early/Late |  |
| 74 | AngHV-1.ORF74.105 | 0.396 | 0.055 | Early/Late |  |
| 85 | AngHV-1.ORF85.114 | 0.388 | 0.007 | Early/Late |  |
| 23* | AngHV-1.ORF23.64 | 0.381 | 0.071 | Early/Late |  |
| 31 | AngHV-1.ORF31.170 | 0.360 | 0.068 | Early/Late |  |
| 116 | AngHV-1.ORF116.183 | 0.359 | 0.002 | Early/Late | Ribonucleotide reductase (large subunit) |
| 76 | AngHV-1.ORF76.107 | 0.344 | 0.024 | Early/Late |  |
| 123 | AngHV-1.ORF123.153 | 0.340 | 0.039 | Early/Late | Deoxyguanosine kinase |
| 98 | AngHV-1.ORF98.128 | 0.339 | 0.120 | Early/Late |  |
| 99* | AngHV-1.ORF99.129 | 0.336 | 0.001 | Early/Late |  |
| 78 | AngHV-1.ORF78.108 | 0.329 | 0.032 | Early/Late | Envelope protein |
| 37* | AngHV-1.ORF37.177 | 0.316 | 0.041 | Early/Late | DNA helicase |
| 5 | AngHV-1.ORF5.49 | 0.313 | 0.064 | Early/Late | Deoxyuridine thriphosphatase |
| 108 | AngHV-1.ORF108.138 | 0.308 | 0.011 | Early/Late | Envelope protein |
| 65 | AngHV-1.ORF65.97 | 0.299 | 0.147 | Early/Late |  |
| 60* | AngHV-1.ORF60.92 | 0.293 | 0.004 | Early/Late |  |
| 91 | AngHV-1.ORF91.121 | 0.291 | 0.009 | Early/Late |  |
| 77* | AnHV.ThymKin.07 | 0.287 | 0.004 | Early/Late | Thymidylate kinase |
| 134 | AngHV-1.ORF134.164 | 0.275 | 0.113 | Early/Late |  |
| 73* | AngHV-1.ORF73.104 | 0.274 | 0.072 | Early/Late |  |
| 38 | AngHV-1.ORF38.75 | 0.271 | 0.023 | Early/Late | Tegument protein |
| 90 | AngHV-1.ORF90.120 | 0.264 | 0.007 | Early/Late | Nucleoside diphosphate kinase |
| 83 | AngHV-1.ORF83.172 | 0.263 | 0.073 | Early/Late | Large tegument protein |
| 21 | AngHV-1.ORF21.182 | 0.262 | 0.058 | Early/Late | Primase |
| 88* | AngHV-1.ORF88.118 | 0.259 | 0.003 | Early/Late |  |
| 29 | AngHV-1.ORF29.185 | 0.259 | 0.025 | Early/Late | Uracil-DNA glycosylase |
| 19 | AngHV-1.ORF19.61 | 0.253 | 0.005 | Early/Late | Tegument protein |
| 89 | AngHV-1.ORF89.119 | 0.251 | 0.058 | Early/Late |  |
| 75 | AngHV-1.ORF75.106 | 0.246 | 0.034 | Early/Late | Thymidylate synthetase |
| 34 | AngHV-1.ORF34.73 | 0.242 | 0.028 | Late | Tegument protein |
| 118 | AngHV-1.ORF118.174 | 0.235 | 0.044 | Late |  |
| 46* | AngHV-1.ORF46.83 | 0.232 | 0.013 | Late |  |
| 53* | AngHV-1.ORF53.87 | 0.227 | 0.064 | Late |  |
| 125 | AngHV-1.ORF125.155 | 0.222 | 0.024 | Late | Envelope protein |
| 61* | AngHV-1.ORF61.93 | 0.216 | 0.019 | Late |  |
| 120* | AngHV-1.ORF120.150 | 0.212 | 0.007 | Late |  |
| 94* | AngHV-1.ORF94.124 | 0.207 | 0.031 | Late |  |
| 43* | AngHV-1.ORF43.80 | 0.206 | 0.072 | Late | Tegument protein |
| 62 | AngHV-1.ORF62.94 | 0.202 | 0.064 | Late |  |
| 33 | AngHV-1.ORF33.72 | 0.202 | 0.050 | Late |  |
| 32* | AngHV-1.ORF32.71 | 0.201 | 0.051 | Late | Tegument protein |
| 71 | AngHV-1.ORF71.103 | 0.199 | 0.023 | Late | Envelope protein |
| 81* | AngHV-1.ORF81.110 | 0.196 | 0.013 | Late | Tegument protein |
| 22 | AngHV-1.ORF22.63 | 0.192 | 0.012 | Late |  |
| 68 | AngHV-1.ORF68.100 | 0.189 | 0.021 | Late |  |
| 79 | AngHV-1.ORF79.187 | 0.188 | 0.027 | Late | Deoxyguanosine kinase |
| 47* | AngHV-1.ORF47.84 | 0.188 | 0.062 | Late |  |
| 40 | AngHV-1.ORF40.77 | 0.185 | 0.038 | Late | Tegument protein |
| 44 | AngHV-1.ORF44.81 | 0.182 | 0.031 | Late |  |
| 20* | AngHV-1.ORF20.62 | 0.179 | 0.002 | Late | Tegument protein |
| 82 | AngHV-1.ORF82.111 | 0.177 | 0.039 | Late |  |
| 11 | AngHV-1.ORF11.53 | 0.176 | 0.012 | Late |  |
| 124* | AngHV-1.ORF124.154 | 0.174 | 0.012 | Late | Tumour necrosis factor receptor domain |
| 119 | AngHV-1.ORF119.149 | 0.170 | 0.028 | Late | Dihydrofolate reductase |
| 102 | AngHV-1.ORF102.132 | 0.166 | 0.008 | Late |  |
| 27* | AngHV-1.ORF27.67 | 0.166 | 0.026 | Late |  |
| 17* | AngHV-1.ORF17.59 | 0.162 | 0.020 | Late | Tegument protein |
| 100 | AngHV-1.ORF100.130 | 0.160 | 0.034 | Late | Capsid protein |
| 12 | AngHV-1.ORF12.54 | 0.154 | 0.029 | Late |  |
| 36* | AngHV-1.ORF36.181 | 0.154 | 0.071 | Late | Capsid triplex protein 2 |
| 114 | AngHV-1.ORF114.144 | 0.153 | 0.025 | Late | Tegument protein |
| 63 | AngHV-1.ORF63.95 | 0.151 | 0.010 | Late |  |
| 26 | AngHV-1.ORF26.66 | 0.147 | 0.062 | Late | Tegument protein |
| 24 | AngHV-1.ORF24.65 | 0.145 | 0.076 | Late | Tegument protein |
| 41* | AngHV-1.ORF41.78 | 0.134 | 0.002 | Late |  |
| 96 | AngHV-1.ORF96.126 | 0.124 | 0.029 | Late | Ribonucleotide reductase (small subunit) |
| 50* | MemProt(KHVORF82).22 | 0.123 | 0.020 | Late |  |
| 97 | AngHV-1.ORF97.127 | 0.116 | 0.001 | Late |  |
| 84* | AngHV-1.ORF84.113 | 0.111 | 0.037 | Late |  |
| 30 | AngHV-1.ORF30.69 | 0.110 | 0.057 | Late | Tegument protein |
| 69* | AngHV-1.ORF69.101 | 0.093 | 0.011 | Late |  |
| 49 | MemProt(KHVORF83).21 | 0.090 | 0.013 | Late | Envelope protein |
| 64* | AngHV-1.ORF64.96 | 0.089 | 0.015 | Late |  |
| 16 | AngHV-1.ORF16.58 | 0.079 | 0.023 | Late | Tegument protein |
| 70 | AngHV-1.ORF70.102 | 0.070 | 0.005 | Late |  |
| 115 | AngHV-1.ORF115.145 | 0.070 | 0.013 | Late | Envelope protein |
| 59 | AngHV-1.ORF59.91 | 0.070 | 0.011 | Late |  |
| 35* | AngHV-1.ORF35.74 | 0.068 | 0.011 | Late | Tegument protein |
| 48 | AngHV-1.ORF48.85 | 0.068 | 0.015 | Late | Capsid protein |
| 57* | AngHV-1.ORF57.178 | 0.066 | 0.022 | Late | Capsid protease-and-scaffolding protein |
| 122* | AngHV-1.ORF122.152 | 0.066 | 0.003 | Late |  |
| 113* | AngHV-1.ORF113.143 | 0.064 | 0.014 | Late |  |
| 54 | AngHV-1.ORF54.88 | 0.063 | 0.035 | Late |  |
| 25* | AngHV-1.ORF25.180 | 0.063 | 0.016 | Late | Interleukin 10 homolog |
| 51 | AngHV-1.ORF51.171 | 0.063 | 0.026 | Late | Envelope protein |
| 103 | AngHV-1.ORF103.133 | 0.057 | 0.012 | Late | Tegument protein |
| 58 | AngHV-1.ORF58.90 | 0.056 | 0.008 | Late |  |
| 104* | AngHV-1.ORF104.134 | 0.050 | 0.007 | Late | Major capsid protein |
| 112 | AngHV-1.ORF112.142 | 0.050 | 0.011 | Late |  |
| 52 | AngHV-1.ORF52.86 | 0.047 | 0.013 | Late |  |
| 121 | AngHV-1.ORF121.175 | 0.047 | 0.005 | Late |  |
| 117 | AngHV-1.ORF117.147 | 0.039 | 0.004 | Late |  |
| 105* | AngHV-1.ORF105.135 | 0.033 | 0.005 | Late |  |
| 126 | AngHV-1.ORF126.156 | 0.031 | 0.008 | Late | Capsid protein |
| 42* | AngHV-1.ORF42.79 | 0.024 | 0.001 | Late | Capsid triplex protein 1 |
| 8 | AngHV-1.ORF8.52 | 0.010 | 0.004 | Late | Envelope protein |
| 95 | AngHV-1.ORF95.125 | 0.010 | 0.000 | Late | Envelope infectious salmon anaemia virus haemagglutinin-esterase protein |

^a^ ORF numbering corresponds to van Beurden *et al*. (2010); ORFs from which the data are potentially compromised by 3’-coterminality are marked with asterisks

^b^ Primer sets correspond to Table S1

^c^ Mean Ri-PAA-values were calculated from two independent experiments

^d^ Standard deviations of Ri-PAA-values were calculated from two independent experiments

^e^ Immediate-early genes were classified on the basis of CHX inhibition experiments; boundaries between early, early-late and late genes are explained in the text

^f^ Gene functions were predicted based on similarity with known functional protein sequences van Beurden *et al*. (2010), and on AngHV1 structural protein analyses by mass spectrometry van Beurden *et al*. (2011)
