# Supplementary material for: Genome-wide gene expression analysis of anguillid herpesvirus 1
Source: BMC Genomics. 2013 Feb 6;14:83. doi: 10.1186/1471-2164-14-83 (PMC3626852; doi:10.1186/1471-2164-14-83)
Supplement: Additional file 3: Table S3 — Sequences and efficiency of qPCR primers targeting 126 AngHV1 ORFs. [file 1471-2164-14-83-S3.docx]

**Table S3 – Sequences and efficiency of qPCR primers targeting 126 AngHV1 ORFs**

| **ORF** | **Primerset** | **Forward start** | **Forward end** | **Forward sequence** | **Reverse start** | **Reverse end** | **Reverse sequence** | **Efficiency (%)** |
| --- | --- | --- | --- | --- | --- | --- | --- | --- |
| ORF1 | AngHV-1.ORF1.45 | 2616 | 2633 | GTGCCTGCGTTCGGATCT | 2671 | 2651 | GGCCCGGGACACTTTATACTG | 94.04 |
| ORF3 | AngHV-1.ORF3.47 | 5329 | 5347 | CGCCTCCTCGTGTTCTTGA | 5385 | 5366 | GCCGACACCTTTCTGAGGAA | 94.98 |
| ORF4 | AngHV-1.ORF4.48 | 6323 | 6339 | TGATGCGAGCCGTGGTT | 6390 | 6371 | AAGACCCGCGAGAAGGAGAA | 93.11 |
| ORF5 | AngHV-1.ORF5.49 | 7494 | 7513 | CCATCGGGTCAGTCATCAGA | 7572 | 7554 | CCCCTTCCCATCCAGATCA | 90.29 |
| ORF6A | AngHV-1.ORF6.169 | 8844 | 8866 | ACTCACAGTCAGGGTTCCGATAG | 8920 | 8900 | CCATGTGGCACCTGATCTTCT | 96.94 |
| ORF8 | AngHV-1.ORF8.52 | 11400 | 11416 | CGGACGGCAGGCTCAAC | 11466 | 11447 | CGCTGATCGCACAGGTACAG | 83.46 |
| ORF10 | AngHV-1.ORF10.184 | 12392 | 12412 | CTGCACGCTGTCTCCATACTG | 12448 | 12432 | GCCCTCTCCGCCCAACT | 94.61 |
| ORF11 | AngHV-1.ORF11.53 | 12750 | 12770 | GGACGCTCAATGGTCACTGTT | 12825 | 12805 | AGTTGGTGCCTGTGAATTGGT | 87.53 |
| ORF12 | AngHV-1.ORF12.54 | 14105 | 14128 | ACACACATCCCACCACTCTACAAT | 14164 | 14147 | TGCCCCAGCCCAGTCTAC | 91.35 |
| ORF13 | AngHV-1.ORF13.55 | 15748 | 15765 | CGTGCTCGGCGAACTCAT | 15808 | 15786 | AAGAGGTGTGTGAACAACGTCAA | 89.81 |
| ORF14 | AngHV-1.ORF14.56 | 16875 | 16893 | AAAAATCCCCACCGACGAA | 16937 | 16918 | CACACCCTGGCTCTGAGCTT | 96.44 |
| ORF15 | AngHV-1.ORF15.57 | 17925 | 17944 | AGGGAGGTCGTGCAGTCAGT | 17982 | 17961 | CTGTGCGGTTGAACATTTTCTC | 98.77 |
| ORF16 | AngHV-1.ORF16.58 | 18584 | 18605 | ATCATTCCCTCCGCAAAAGTAC | 18642 | 18622 | GCATAACGATGCCTCCAGAAG | 91.88 |
| ORF17 | AngHV-1.ORF17.59 | 19457 | 19476 | GCCAAAAACATGCGATACCA | 19529 | 19510 | GCGTGATACTGGACGTGGAA | 84.78 |
| ORF18 | AngHV-1.ORF18.60 | 20416 | 20431 | TCGCAACCCGGCACTT | 20482 | 20464 | AGGCGCTGGTTAGGTGCTT | 93.60 |
| ORF19 | AngHV-1.ORF19.61 | 26530 | 26551 | TCGTGGTTGAAGCAGGTACAGA | 26613 | 26594 | CGGTGACGGAATGTGTAACG | 86.89 |
| ORF20 | AngHV-1.ORF20.62 | 30629 | 30646 | CAGCAGATCCGCCCTCAA | 30689 | 30669 | CGCAACTTGTAACGCCTTTTC | 88.93 |
| ORF21 | AngHV-1.ORF21.182 | 31273 | 31294 | GCCGAAGAGTACGAGGAGATGA | 31334 | 31316 | CGCACGCTGAGCAGAGATC | 93.30 |
| ORF22 | AngHV-1.ORF22.63 | 33745 | 33767 | TGGATATGTGTTCTGCCTGTTTG | 33814 | 33792 | CCCTTTAATCGGTGCTCAATAGA | 86.13 |
| ORF23 | AngHV-1.ORF23.64 | 35766 | 35784 | CGAAACGGCAGCTCTGATC | 35826 | 35806 | TGACACGGTTTTGGCTACACA | 91.02 |
| ORF24 | AngHV-1.ORF24.65 | 37637 | 37658 | GAACACGTAGAGGCGTTTCTCA | 37699 | 37678 | CCTGCGACTGTGGAGAAGTTCT | 88.24 |
| ORF25 | AngHV-1.ORF25.180 | 38198 | 38217 | CACCACACCAAATGCGTCAT | 38253 | 38235 | AGCAAGTCGGCACCTTCCT | 97.38 |
| ORF26 | AngHV-1.ORF26.66 | 39701 | 39721 | CCAACGGTCTGTTCCAACATC | 39770 | 39751 | GCTCGCAGCTCTCTTTTGGT | 93.41 |
| ORF27 | AngHV-1.ORF27.67 | 40359 | 40377 | CTTCCAGCCGAGGCTACGT | 40423 | 40403 | GGTGAGAGACAGCAGCGATGT | 90.31 |
| ORF28 | AngHV-1.ORF28.68 | 41611 | 41629 | TGGGCTATGGAGGGATGGA | 41668 | 41649 | ATGTGAGCGAGGTCGGATTT | 85.34 |
| ORF29 | AngHV-1.ORF29.185 | 42405 | 42425 | CGCTGAAAACAACTGCTGGTT | 42469 | 42450 | TCGCACAGGTTGCTCTCAAT | 96.42 |
| ORF30 | AngHV-1.ORF30.69 | 46078 | 46100 | CTGTCAGACAATCGTCGACAAAC | 46166 | 46143 | CCATCGTGCAGTAGTAAACCACAT | 90.94 |
| ORF31 | AngHV-1.ORF31.170 | 48507 | 48526 | GAGTCTACGGCACGGTGGTT | 48576 | 48554 | AACGCAGAATCTTGTTGAAAAGG | 94.71 |
| ORF32 | AngHV-1.ORF32.71 | 48840 | 48855 | CCCCCGCCGACAAACT | 48909 | 48887 | TCTACTGCCCAAGAGATGCTGAT | 86.56 |
| ORF33 | AngHV-1.ORF33.72 | 49516 | 49537 | CACCACACACAACAGCAATGAC | 49589 | 49571 | CGAGCACCGTGATCGTGAT | 84.52 |
| ORF34 | AngHV-1.ORF34.73 | 51416 | 51438 | AAAACACGAACCTCCACCTACAC | 51492 | 51467 | CTTATACATATCACCGAAGGCTTTGA | 88.74 |
| ORF35 | AngHV-1.ORF35.74 | 56128 | 56144 | CCTTCTGCCCGCATGGT | 56200 | 56176 | ACAGTCTCCGAAAGTGAAGATAGGA | 94.91 |
| ORF36 | AngHV-1.ORF36.181 | 56939 | 56958 | TCTGCCCACCACGTACTTGA | 56998 | 56981 | GCGGCGACACGTCTGATC | 90.06 |
| ORF37 | AngHV-1.ORF37.177 | 58658 | 58678 | ACGCCAGCCACATACTTGAAC | 58728 | 58708 | CACGCTTGTCCCAAAATCATT | 95.73 |
| ORF38 | AngHV-1.ORF38.75 | 60121 | 60140 | GGCAGCAACCATCATCCTTT | 60180 | 60161 | TTGGGAGCCGAAGTATTGGA | 94.57 |
| ORF39 | AngHV-1.ORF39.76 | 61881 | 61901 | CAACCCCACATCAGAGTCCAA | 61955 | 61932 | TGCGTCAACAAGAGTGTAGTCCAT | 98.37 |
| ORF40 | AngHV-1.ORF40.77 | 63135 | 63156 | CAAACCACAAGAGCCCTACCAA | 63216 | 63195 | TGTTACGGACTCGATGGTTCTG | 92.25 |
| ORF41 | AngHV-1.ORF41.78 | 66786 | 66805 | TGGTGTAAAAACGCCGTGAA | 66846 | 66827 | GGTGTCCCAGGCAATCTTGA | 99.88 |
| ORF42 | AngHV-1.ORF42.79 | 67831 | 67851 | CACGTCACAAAACACGGAGAA | 67887 | 67872 | CGGCTGCGGGTTGAGA | 94.18 |
| ORF43 | AngHV-1.ORF43.80 | 68644 | 68626 | GCAGATTGGTCGCCCTTTT | 68686 | 68666 | CTTGCCACAACGGTTACCATT | 91.17 |
| ORF44 | AngHV-1.ORF44.81 | 71525 | 71542 | CAGCCGTCCGCCTCATAA | 71585 | 71566 | GGCTTCTGTGCCCAACTTTC | 95.85 |
| ORF45 | AngHV-1.ORF45.82 | 72519 | 72541 | CAGCACCGAAACAGTATCTTGTG | 72582 | 72563 | CTGGACGCAATTTTGGGAAT | 96.70 |
| ORF46 | AngHV-1.ORF46.83 | 75500 | 75519 | TCGCCACTGCTTTCAGAAGA | 75559 | 75540 | CCCTTCCCTCAGACCTTTCC | 91.74 |
| ORF47 | AngHV-1.ORF47.84 | 76513 | 76533 | AACTCTGCTGCAAGGGTGAAG | 76573 | 76551 | TCAGTACAGCCCCAATTACATCA | 96.07 |
| ORF48 | AngHV-1.ORF48.85 | 78539 | 78559 | TGACCCCTAGCACGTTTGATT | 78597 | 78580 | CGACGACGCCTCTTCCAT | 94.43 |
| ORF49 | MemProt(KHVORF83).21 | 80462 | 80481 | GGACGGACTGTTTGGCATGT | 80526 | 80508 | CCACGCACCACACAATCAA | 91.43 |
| ORF50 | MemProt(KHVORF82).22 | 81638 | 81656 | CTGCCATGAAACCCAAACG | 81705 | 81685 | TCTGTAGTCGCCCCAAACTTG | 91.21 |
| ORF51 | AngHV-1.ORF51.171 | 82184 | 82202 | GGAACCTGGTGGCATCAGA | 82257 | 82237 | TGCACAAAGAGACCGACAACA | 93.91 |
| ORF52 | AngHV-1.ORF52.86 | 82897 | 82918 | CGAAGGGAAAGAAAGGATTCAA | 82971 | 82953 | CGAGGGTTGGTTGCTGAGA | 91.65 |
| ORF53 | AngHV-1.ORF53.87 | 84599 | 84622 | AAGCTACTACTCCTCCCACACCAA | 84656 | 84639 | TTGCAGGTCCACGTTCCA | 96.58 |
| ORF54 | AngHV-1.ORF54.88 | 85783 | 85808 | TGAGAGTTTAGAATCCGCTGAGTACA | 85866 | 85848 | TCACCCCGTTCCTCTGGTT | 94.73 |
| ORF55 | AngHV-1.ORF55.179 | 86856 | 86879 | TTCGCTCGTACAGGTTCAATAGAG | 86917 | 86897 | ACAGCCGACTTTGTTGGAAGA | 93.57 |
| ORF56 | AngHV-1.ORF56.89 | 91802 | 91820 | TCCCCACACCCGAAAAGAT | 91882 | 91858 | CCAACACAATGACTTGACATATCCA | 93.31 |
| ORF57 | AngHV-1.ORF57.178 | 93318 | 93333 | ACCCGCTCGCCCTGAT | 93381 | 93360 | TCTACTCCGTGCTGACCAAAGA | 93.91 |
| ORF58 | AngHV-1.ORF58.90 | 94485 | 94506 | CGGGTTAGGGACTCTGTGAAAG | 94542 | 94524 | GACGCTCGGCACAAAGTGT | 96.13 |
| ORF59 | AngHV-1.ORF59.91 | 95208 | 95228 | CGCTCAGACAGTTTGGAAAGG | 95268 | 95249 | CCCCTGGAAGCAGTGAAGAA | 94.78 |
| ORF60 | AngHV-1.ORF60.92 | 95785 | 95802 | GCTCCAACCTGCGTTTCG | 95852 | 95832 | AAAGCCCGTTGAACCATTTTC | 94.64 |
| ORF61 | AngHV-1.ORF61.93 | 97656 | 97673 | CGGCGACACGTCCATTTC | 97727 | 97708 | CACCCCAGAGATGTGCAACA | 93.91 |
| ORF62 | AngHV-1.ORF62.94 | 98459 | 98482 | TGCGAGTTGAGAGATTGTAAAAGC | 98519 | 98501 | AATGCCAAGGCGTGTTGAG | 96.13 |
| ORF63 | AngHV-1.ORF63.95 | 98947 | 98964 | ACGACCCGAACCCGAGAT | 99002 | 98982 | CCCTTTGTAAAGCAGCGTGTT | 94.38 |
| ORF64 | AngHV-1.ORF64.96 | 99286 | 99306 | GGGTCTGAACGGAACCATTTT | 99350 | 99332 | GAGGAGCCGCAGCTAACCT | 87.58 |
| ORF65 | AngHV-1.ORF65.97 | 101577 | 101598 | ACATTTGGTATCGCTGCTCTCA | 101636 | 101618 | CAATGCACGTCCGAGGAAT | 98.76 |
| ORF66 | AngHV-1.ORF66.98 | 103453 | 103474 | CCCAACTCCGCAATCAGTTTAA | 103517 | 103495 | TCTTTCTCCTCATCACCTTTCGT | 99.02 |
| ORF67 | AngHV-1.ORF67.99 | 105856 | 105877 | GTCTCTGCGTGTTCAGCAAGTC | 105921 | 105902 | CTATCGCCAAGCACCTGACA | 85.59 |
| ORF68 | AngHV-1.ORF68.100 | 108907 | 108926 | TGCGTTTCTTTTCGGGAGAT | 108979 | 108957 | CAGCAGCTAAAATTGGACGACAT | 95.87 |
| ORF69 | AngHV-1.ORF69.101 | 109524 | 109547 | CATCAGATTCCAGAGCTTGTTGTC | 109594 | 109578 | CCTTGCCCACGCACTTG | 98.37 |
| ORF70 | AngHV-1.ORF70.102 | 109998 | 110016 | TCCCACAGCTTCGCATCAA | 110053 | 110032 | GCTCGTTTTCTGACCGAAGATT | 97.97 |
| ORF71 | AngHV-1.ORF71.103 | 110422 | 110442 | CGTCCTCCGGTCTAAATGTTG | 110481 | 110462 | GCGATGGACTGGATGACACA | 92.69 |
| ORF73 | AngHV-1.ORF73.104 | 112062 | 112079 | CTCCGTCTCCCGCTTTGA | 112128 | 112109 | GCTCTGGATGCCATCGACTT | 99.75 |
| ORF74 | AngHV-1.ORF74.105 | 112649 | 112667 | TAGCAACGGCGCTCATGAT | 112702 | 112683 | GGCTGTGGGTGGTAGAAACG | 92.12 |
| ORF75 | AngHV-1.ORF75.106 | 116004 | 116023 | CCTCCGTGTCACCTCTTTGC | 116072 | 116052 | CGTTATCGTCCCGTGTTGGTA | 98.77 |
| ORF76 | AngHV-1.ORF76.107 | 116851 | 116869 | CAAACCACGGCGAAATGTT | 116910 | 116891 | GCGTCATTGTTGGAGAGCAA | 98.90 |
| ORF77 | AnHV.ThymKin.07 | 118110 | 118126 | GGACGGCGGTGATGGAT | 118176 | 118155 | AGGACTGGAAAAAGCACGTTGT | 103.08 |
| ORF78 | AngHV-1.ORF78.108 | 118368 | 118388 | GATGGTGTTTGCGGTGACAGT | 118437 | 118414 | GTTCTAGGCGGGCTTATTTTATTG | 99.42 |
| ORF79 | AngHV-1.ORF79.187 | 119425 | 119444 | GAGAAGGAGCGGAAGATGTC | 119500 | 119481 | GGGTACATCGGGTTCATTTC | 95.62 |
| ORF80 | AngHV-1.ORF80.109 | 121784 | 121802 | CCCCACTCTTCACCGACAA | 121842 | 121822 | CACGACACTCCGCTGTTGAAT | 93.67 |
| ORF81 | AngHV-1.ORF81.110 | 122991 | 123008 | ACGCCTCCACCCAACAGA | 123051 | 123031 | CCGTGCTCAACACCATCAAAC | 91.12 |
| ORF82 | AngHV-1.ORF82.111 | 123481 | 123500 | TTTCCACGGTTTGAGCCATT | 123540 | 123521 | CAGAACAGCGGTCGGTAGGA | 100.39 |
| ORF83 | AngHV-1.ORF83.172 | 128294 | 128316 | CCGGACGAGTTGGTCATAGTAGA | 128374 | 128351 | CCAGACCATATCGAAAAAGTTGAA | 91.03 |
| ORF84 | AngHV-1.ORF84.113 | 136300 | 136318 | CACGAGGGTTGGCACAGAA | 136373 | 136350 | TTGAGCAGAATCCAGAAAGATTTG | 96.37 |
| ORF85 | AngHV-1.ORF85.114 | 137116 | 137132 | CATGGCGGGCTGAGACA | 137171 | 137152 | TGGACCAGCAAGTGCAGACT | 94.71 |
| ORF86 | AngHV-1.ORF86.115 | 141018 | 141037 | AAAGGATCGTGGCGAAAATG | 141076 | 141054 | TTGTTCCTCAGCACTGTCATGTT | 91.31 |
| ORF87 | AngHV-1.ORF87.117 | 144750 | 144768 | GAGACGGACTGGCGGAGAT | 144825 | 144808 | CGTTCGGGTGGGTTCAGA | 97.42 |
| ORF88 | AngHV-1.ORF88.118 | 145599 | 145618 | AGCCGAAGTGCCAGAGAATG | 145686 | 145668 | GCCGTACCCCTCACACACA | 95.79 |
| ORF89 | AngHV-1.ORF89.119 | 148223 | 148242 | CCGCCTCCAAATAGGTTTCC | 148295 | 148276 | CCCCGACAACACTGCTATGA | 101.00 |
| ORF90 | AngHV-1.ORF90.120 | 149070 | 149086 | GCCAGGAGCAGCGACAA | 149124 | 149104 | GCAGTTGGTGAGGTTGATGGT | 99.86 |
| ORF91 | AngHV-1.ORF91.121 | 150258 | 150276 | GCCAGTCACCGAAGCGATT | 150316 | 150297 | GCCACTCCGAGTCCTTCAAC | 102.98 |
| ORF92 | AngHV-1.ORF92.122 | 153335 | 153354 | CCAGTCGCAGCAGATTCTCA | 153394 | 153378 | GGCGGCGTCAACAAATG | 90.27 |
| ORF93 | AngHV-1.ORF93.123 | 155880 | 155903 | TGGTTTGTTGGTTGGTCTTACATT | 155947 | 155925 | TCATGTACTGTAGCACGGATGGT | 89.10 |
| ORF94 | AngHV-1.ORF94.124 | 157670 | 157692 | CAGAGTGAGAGTCCGCTACAACA | 157729 | 157712 | AACGGGCAGCCTGGATCT | 92.42 |
| ORF95 | AngHV-1.ORF95.125 | 158337 | 158356 | CACCAAACAACCCAGCACAA | 158400 | 158381 | GCCGTTTGACAAGCAGTTCA | 103.50 |
| ORF96 | AngHV-1.ORF96.126 | 160080 | 160099 | CAAAAGAGGCGTTGGTGTCA | 160144 | 160128 | GCCGTGCATCGCAAAGA | 103.87 |
| ORF97 | AngHV-1.ORF97.127 | 160741 | 160762 | TGGAGAGAGAGTCTGCGATGTC | 160815 | 160799 | GGATGGCGGAGGAGGAA | 100.48 |
| ORF98 | AngHV-1.ORF98.128 | 164974 | 164993 | CTGCGACGCTCCTTCTACCT | 165036 | 165012 | CTTGTCAAATGTTCTGGTCTTTCTG | 96.82 |
| ORF99 | AngHV-1.ORF99.129 | 167865 | 167888 | GGTGTACGCTGAGCAACTATTGTG | 167941 | 167921 | GAACCAGACCCGACAGAGTCA | 96.04 |
| ORF100 | AngHV-1.ORF100.130 | 169658 | 169680 | CCCAATGCTCTTCTGTCTACGTT | 169732 | 169710 | CAACAGCCTCCTATCCAAACTGA | 101.50 |
| ORF101 | AngHV-1.ORF101.131 | 170410 | 170428 | CGGGATGGCGTCTATTGGT | 170469 | 170451 | AGGGATGTGGGTCCATTCC | 102.44 |
| ORF102 | AngHV-1.ORF102.132 | 172821 | 172836 | TCGTGGGCGGTTTGCT | 172888 | 172869 | TCAGGCGGTGTTCTGCATTA | 101.63 |
| ORF103 | AngHV-1.ORF103.133 | 173613 | 173633 | TTTGGTCGCTGCTTAGAGGAA | 173697 | 173676 | CGTCCACGTCTTGAATTTGTTC | 100.61 |
| ORF104 | AngHV-1.ORF104.134 | 176725 | 176746 | CAGTGGTCCCTGATGTCTATGC | 176793 | 176774 | CATCACACCCTGACCCCATT | 88.81 |
| ORF105 | AngHV-1.ORF105.135 | 177954 | 177972 | GCAGCTCAAACCGGTGTCA | 178028 | 178013 | GGCGGCCACGTTCTTG | 98.45 |
| ORF106 | AngHV-1.ORF106.136 | 182577 | 182597 | GCGAGTATTTGGAAGCTGGAA | 182639 | 182623 | GCTCGAACGCAGCGAAA | 101.28 |
| ORF107 | AngHV-1.ORF107.137 | 185268 | 185286 | GCCAACCAGCGAACCAATA | 185330 | 185308 | AAACTGCAAAGCCCTTAAACACA | 88.56 |
| ORF108 | AngHV-1.ORF108.138 | 189493 | 189512 | CGTGTTACCGCAGGTCATG | 189554 | 159535 | CGCTCGTTCTCCAGCTCATC | 93.95 |
| ORF109 | AngHV-1.ORF109.173 | 191092 | 191109 | CGACCACGGCATGTTTCA | 191151 | 191131 | TCGACAACATCACCGTCAATC | 95.56 |
| ORF110 | AngHV-1.ORF110.140 | 195250 | 195268 | CCTCTGCTGGGCAAGGTTT | 195312 | 195294 | CACTGTCACGTCGCCACAA | 94.63 |
| ORF111 | AngHV-1.ORF111.141 | 199422 | 199440 | GCAACGCAGATCGGTTTTG | 199479 | 199460 | GCGGGATGGAGGTGTTAGAG | 99.77 |
| ORF112 | AngHV-1.ORF112.142 | 199902 | 199925 | GTGATGATAGTTTGTGGCGAACTT | 199991 | 199966 | AAAACTAACAAAGAGGCAGATGATGA | 97.17 |
| ORF113 | AngHV-1.ORF113.143 | 201922 | 201938 | GCCACGGTTCGGGTGTT | 201980 | 201961 | CCATCCGCCCGTATGTTTAC | 99.07 |
| ORF114 | AngHV-1.ORF114.144 | 202826 | 202845 | AACTTCCACGCAGCCTTCAG | 202896 | 202878 | CCCGAGTTCCCCCAATCTT | 99.42 |
| ORF115 | AngHV-1.ORF115.145 | 203060 | 203079 | GACGGGTTGCGTGTTTGATT | 203131 | 203111 | CCCAGACAGTCGTTGACACAA | 94.17 |
| ORF116 | AngHV-1.ORF116.183 | 204300 | 204318 | GAGCCAGGAGCAGGGAGTT | 204367 | 204347 | GCACGACTGGACCTCTCTGAA | 95.21 |
| ORF117 | AngHV-1.ORF117.147 | 206771 | 206787 | GCAGAGCCGCCGAGAAC | 206824 | 206805 | GATCGTCCTCTTGGGAATGC | 101.14 |
| ORF118 | AngHV-1.ORF118.174 | 207501 | 207518 | GCTGGTGTGGCTGCAACA | 207560 | 207538 | CACTCCTGTAGTCGCAGTCAGAA | 96.36 |
| ORF119 | AngHV-1.ORF119.149 | 207889 | 207907 | TGGACGGTGGAGCAAGTTC | 207957 | 207935 | CCTAGTTTTATCGGCAGGTGACA | 88.28 |
| ORF120 | AngHV-1.ORF120.150 | 209329 | 209349 | GACTGACGGAACCAGCTATCG | 209398 | 209383 | GCGGCGTTGGGACCAT | 92.47 |
| ORF121 | AngHV-1.ORF121.175 | 212503 | 212521 | GGCAAGGTGGATCAACGAA | 212558 | 212541 | ACTGCGGGTCAGCCTCAA | 96.42 |
| ORF122 | AngHV-1.ORF122.152 | 213357 | 213372 | CACCAACGCCGCAACA | 213411 | 213391 | TGTGAAATGGCCCAAGGTAGA | 95.63 |
| ORF123 | AngHV-1.ORF123.153 | 213927 | 213946 | CCCTGAACCCGTCAACTTGT | 214012 | 213990 | GAAAGGTGTGCTCGTCTTGATCT | 97.95 |
| ORF124 | AngHV-1.ORF124.154 | 214838 | 214857 | TGTCCAGCAGGCACTCATGT | 214904 | 214888 | GACACGCACCGCATTCG | 101.01 |
| ORF125 | AngHV-1.ORF125.155 | 218407 | 218426 | AGGATCGGCTTCACAATGGT | 218477 | 218456 | AACATTTGAAAGGCGAGAACGT | 98.16 |
| ORF126 | AngHV-1.ORF126.156 | 220658 | 220675 | ACGCTGCGGTGGTGAAAG | 220724 | 220705 | GCCTCCAGATCGGTCTGATC | 90.54 |
| ORF127 | AngHV-1.ORF127.157 | 221505 | 221524 | GGGTACATCGGCTCGTCTGT | 221569 | 221550 | CGAAAGATCCCAACGTGCAT | 92.66 |
| ORF130 | AngHV-1.ORF130.160 | 225333 | 225354 | TTGCGGGTTTCTGTAGACATCA | 225404 | 225382 | AGAATGGGTCAAGATCACACACA | 92.13 |
| ORF131 | AngHV-1.ORF131.176 | 227777 | 227800 | AGTACAATTCTCCCGAGCTGTCTT | 227839 | 227818 | TCAGACCACTGGCTACGACAGA | 84.37 |
| ORF134 | AngHV-1.ORF134.164 | 233762 | 233783 | CGTTCGTAGCTTTGATGGAGAA | 233824 | 233804 | CCAGAGGGACCGTGAAGAACT | 98.89 |
